# Geomechanical log responses and identification of fractures in tight sandstone, West Sichuan Xinchang Gas Field

**DOI:** 10.1038/s41598-022-19995-8

**Published:** 2022-09-15

**Authors:** Jingling Xu, Ruotao Wang, Ling Zan, Xiaoguang Wang, Jiaqing Huo

**Affiliations:** 1 School of Geophysics and Information Technology, China University of Geosciences (Beijing), Beijing, 100083 China; 2 Key Laboratory of Geo-Detection (China University of Geosciences, Beijing), Ministry of Education, Beijing, 100083 China; 3 Research Institute of Petroleum Exploration & Development, East China Company, SINOPEC, Nanjing, 210019 China

**Keywords:** Geophysics, Acoustics

## Abstract

Natural fractures provide important reservoir space and migration channels for oil and gas in tight reservoirs. Moreover, they are key factors controlling the high yield of tight oil and gas. Accordingly, methods to identify and characterize fractures are essential; however, conventional well-logging data are not ideal for such purposes. To this end, our study proposed an efficient method for identifying and characterizing fractures. First, core observations, core sample test analysis, numerical simulations, core calibration of borehole image logs, and borehole image log calibration of conventional logs were performed to identify sensitive log curves and log response characteristics of fractures. Second, we analyzed the response characteristics and differences in the log and properties of tight sandstone with and without fractures. Third, logging eigenvalue (EvF) and rock physical eigenvalue (MvF) models were constructed to determine different causes of fractures in tight sandstone. Finally, the two models were applied to identify and characterize fractures in the tight sandstone reservoirs in the West Sichuan Xinchang gas field, China. It was found that the effect of using the logging eigenvalue and rock physical eigenvalue models to identify fractures was similar to that observed using an image log. Overall, different fractures were accurately identified and characterized, indicating that the proposed method efficiently identifies and characterizes fractures in tight sandstone, ultimately advancing the research of fine reservoir evaluation and fracture theory.

## Introduction

Unconventional tight hydrocarbons have been major candidates for current and future hydrocarbon production, and tight rocks are the focus of current and future research. Tight rocks are highly heterogeneous and tight, with poor permeability^1,2^. Natural fractures are key reservoirs and migration channels for tight oil and gas systems, and hydraulic fracturing is a revolutionary technology widely applied to develop tight hydrocarbon reservoirs^2–4^. The role of fractures, especially natural fractures, in improving production determines the ability to produce tight oil and gas^2,5–8^. Therefore, it is of great significance to study the natural fractures of tight rock. Conventional well-logging can help identify and evaluate reservoirs, hydrocarbons, and fractures. Full-borehole microresistivity imaging (FMI) can be performed to estimate fractures and quantify fracture parameters (e.g., fracture porosity, fracture density, and fracture dip). Although some studies have already used FMI data to study fracture parameters^9–12^, FMI is very expensive, related data are limited, and some wells completely lack FMI data. Conventional well-logging data can also be used to evaluate fracture porosity, while the finite element method (FEM) has been previously utilized to quantify fracture parameters by analyzing dual laterolog resistivity (RLLD, i.e., RT) responses^13^. To date, most studies have examined fractures using conventional logging data^14–22^.

In this context, Wang^23^ introduced a discrete fracture network (DFN) model based on core fracture measurements and borehole image log interpretation data. The author used multivariate statistical and random interpolation methods to model and forecast fracture parameters. Wang et al.^24^ further suggested that natural underground reservoir fractures can be studied in terms of their structural characteristics via tectonic stress field simulation technology. They quantitatively characterized and predicted the fracture parameters based on the rock failure criterion and strain energy density. Aghli et al.^25^ have applied conventional petrophysical logs and their correlation with borehole image logs to identify fracture zones using the differentiation method.

Furthermore, numerous other studies (Lai et al. 2020, Fan et al. 2016, Ding et al. 2017, He et al. 2020, Tian et al. 2021, Ren et al. 2020) have focused on the identification and evaluation of carbonate fractures^21,26–30^. Despite promising results, these methods cannot be applied to tight sandstone reservoirs, prompting the development of new methods for evaluating fracture parameters such as fracture porosity. Generally, conventional well-logging data (acoustic and density) are sensitive to fractures in tight reservoirs^31^. On this basis, conventional well-logging data can be used to identify and evaluate fractures in tight reservoirs. Besides the limitations above, the borehole image log data are generally limited, thereby motivating the introduction of fracture modeling, especially when the data are limited. To date, the conventional logging and rock physical response characteristics of fractures have been described only for certain rock types. Moreover, methods for identifying and characterizing fractures are scanty, and the data stemming from new logging technologies are limited.

The main aim of this study was to explore the log and petrophysical response characteristics and differences of fractures with various geological geneses. To this end, we introduced a method based on core and conventional logging data. The method was used to identify fractures with different geological geneses in tight reservoirs in the western Sichuan Basin. The target layer was a tight sandstone reservoir with mainly low-angle fractures^32–34^. We performed (a) numerical simulations of the log response of fractures, (b) analyzed the log response characteristics of fractures in tight sandstone, (c) determined the log response characteristics and geomechanical properties of fractures, and (e) elucidated the differences in geomechanical log responses to fractured rock. Two new models (eigenvalue functions EvF and MvF) were proposed to efficiently distinguish between different types of fractures in the western Sichuan Basin as a case study, using conventional logs and rock physical responses.

## Geological background

**Figure 1:**
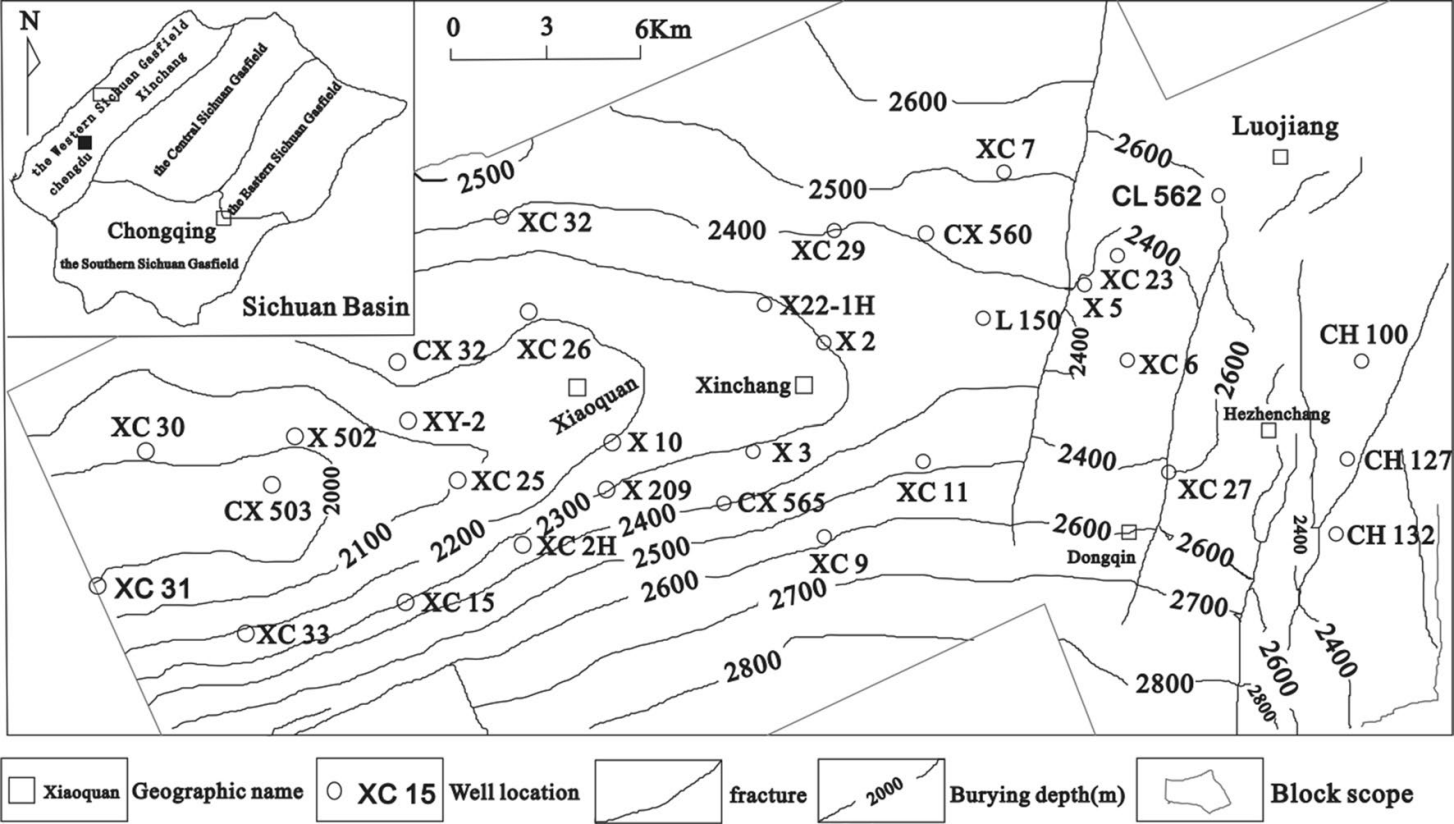
Study area and structural characteristics.

**Figure 2:**
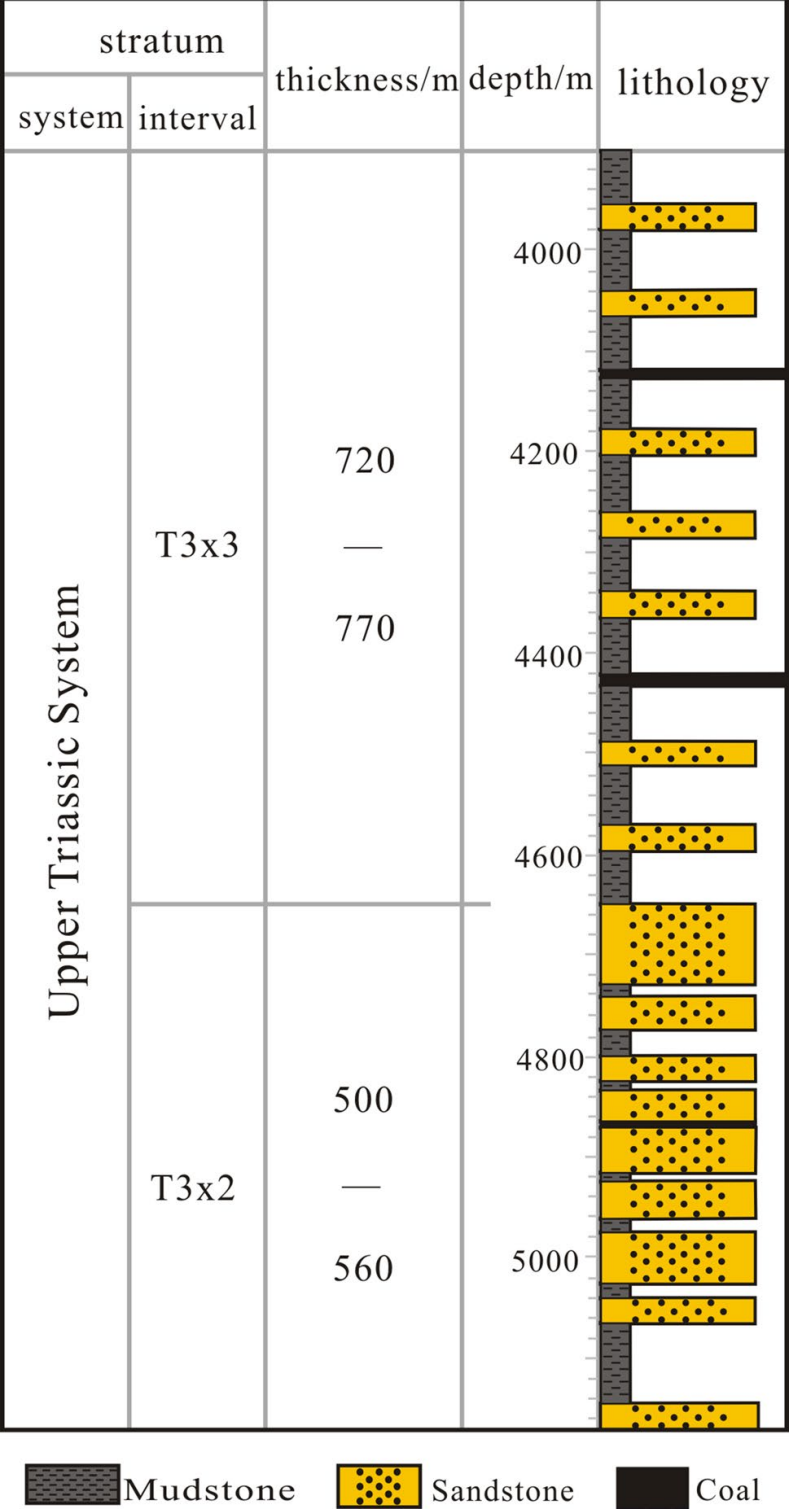
Lithological characteristics of the target layer in the study area.

The West Sichuan Xinchang gas field is located ~ 20 km north of Deyang City (the Sichuan Province). From a tectonic perspective, it is located in the middle of the Mianzhu-Yanting ENE-trending large uplift belt in the middle of the western Sichuan Depression (Fig. 1). The study area includes Middle Triassic marine limestone. Since the Late Triassic, the strata in the western Sichuan area have gradually transitioned into continental deposits. The area is filled with the upper Triassic Xu 1st (T3X1) sea-land transitional facies strata, Xu 2nd (T3X2) member, Xu 3rd (T3X3) member, Xu 4th (T3X4) member, and Xu 5th (T3X5) member, which represent continental clastic and coal-measure strata (Fig. 2). Of them, T3X1, T3X3, and T3X5 are dominated by source rocks, while T3X2 and T3X4 are the main producing layers of the Xinchang gas field. Note that the T3X2 is the layer of interest in this study. The burial depth of T3X2 is 4500–5300 m; it comprises countless thick sandstones and is characterized by interbedded thick sandstones and thin mudstones with a sand-mud ratio of ~ 3:1^35^. The Xinchang area is dominated by braided river delta deposits, while T3X2 mainly consists of delta front deposits. The pay zone mainly comprises distributary channels and estuary-dam microfacies^36^. T3X2 is characterized by well-developed fractures, fairly developed tensile fractures in the sandstone, and fairly developed shear fractures in the mudstone. The development effectiveness of sandstone fractures was higher than that of mudstone fractures^36^. The range of the T3X2 reservoir porosity is 0.5%–6%, average porosity is 3.5%, and permeability is generally < 0.1 × 10^–3^ μm^2^. However, under the same conditions, the permeability of fractured reservoirs is higher than that of non-fractured reservoirs. Overall, there are three types of effective reservoirs: pore, fracture–pore, and fracture type^36^.

## Numerical simulation of fractured and non-fractured logging response

**Figure 3:**
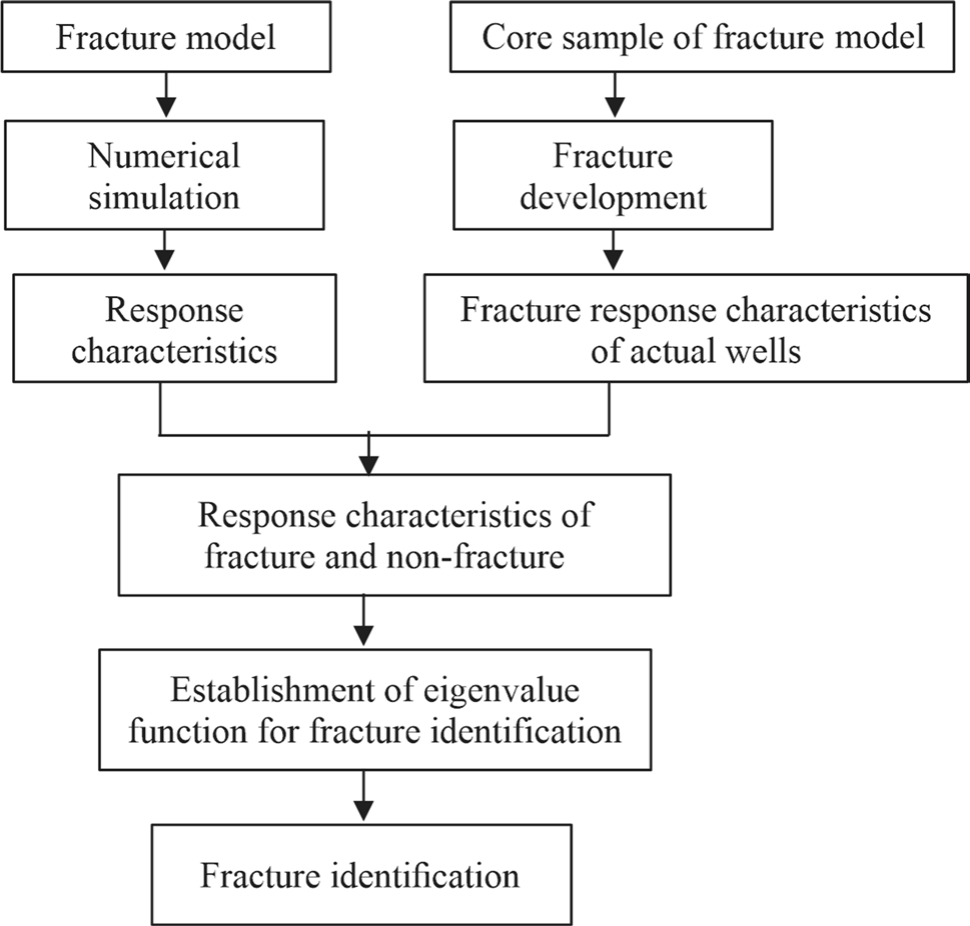
General methodology and technical routes of the study.

The methodology applied to identify fractures is shown in Fig. 3. Note that the fracture identification methodology was formulated according to the technical workflow. In this context, the effective difference method was used for numerical simulations. The derivation process of the finite-difference method in the sound field simulation is described below.

All the wave trains and problems analyzed and simulated in this study were based on the online elastic dynamics theory. Extensive physical experiments in the past 100 years have confirmed the accuracy of elastic wave theory. The finite-difference method is mainly used to simulate the sound field. By assuming that the object is continuous and the deformation is linearly elastic, the motion equation of the particle movement in the object can be formalized as follows^37^:

1 $$\rho \frac{{\partial }^{2}{u}_{i}}{\partial {t}^{2}}=\frac{\partial {\tau }_{ij}}{\partial {x}_{j}}+{F}_{i}$$

where ${u}_{i}$ is the displacement of the object, ${\tau }_{ij}$ is the stress, and ${F}_{i}$ is the volume density, *i*, *j* = 1, 2, 3. The first derivative of displacement is velocity, implying that Eq. (1) can be rewritten for velocity as Eq. (2):

2 $${\tau }_{i j}=\lambda \theta {\delta }_{i j}+2 \mu {\varepsilon }_{i j}$$

where $$\theta = \nabla \cdot \overset{\lower0.5em\hbox{$\smash{\scriptscriptstyle\rightharpoonup}$}}{U}$$ reflects the volumetric strain, $$\overset{\lower0.5em\hbox{$\smash{\scriptscriptstyle\rightharpoonup}$}}{U}$$ is the displacement vector, $$\lambda$$ and $$\mu$$ are the Lame coefficients, $${\varepsilon }_{i j}$$ stands for strain, and $${\delta }_{ij}$$ represents the Kronecker delta (when *i* is not equal to *j*, the value is 0; when *i* is equal to *j*, the value is 1).

Given the application of the axisymmetric model, the three-dimensional model can be simplified as a two-dimensional model after derivation. Thus, the problem can be solved using two-dimensional motion and constitutive equations, as shown by Eq. (3):

3 $$\begin{aligned} \frac{{\partial v_{r} }}{\partial t} & = \frac{1}{\rho }\left( {\frac{1}{r}\tau_{rr} + \frac{{\partial \tau_{rr} }}{\partial r} + \frac{{\partial \tau_{rz} }}{\partial z}} \right) \\ \frac{{\partial v_{z} }}{\partial t} & = \frac{1}{\rho }\left( {\frac{1}{r}\tau_{rz} + \frac{{\partial \tau_{rz} }}{\partial r} + \frac{{\partial \tau_{zz} }}{\partial z}} \right) \\ \frac{{\partial \tau_{rr} }}{\partial t} & = \rho c_{p}^{2} \frac{{\partial v_{r} }}{\partial r} + \rho \left( {c_{p}^{2} - 2c_{s}^{2} } \right)\left( {\frac{{v_{r} }}{r} + \frac{{\partial v_{z} }}{\partial z}} \right) \\ \frac{{\partial \tau_{zz} }}{\partial t} & = \rho c_{p}^{2} \frac{{\partial v_{z} }}{\partial z} + \rho \left( {c_{p}^{2} - 2c_{s}^{2} } \right)\left( {\frac{{v_{r} }}{r} + \frac{{\partial v_{r} }}{\partial r}} \right) \\ \frac{{\partial \tau_{rz} }}{\partial t} & = \rho c_{s}^{2} \left( {\frac{{\partial v_{r} }}{\partial z} + \frac{{\partial v_{z} }}{\partial r}} \right) \\ \end{aligned}$$

After derivation, the derivative of the velocity with respect to the *r* and *z* directions is finally obtained using Eq. (4):

4 $$\begin{aligned} D_{r}^{ - } v_{r}^{{n + \frac{1}{2}}} & = \mathop \sum \limits_{l = 1}^{\frac{L}{2}} a_{l}^{r} \left[ {v_{r}^{{n + \frac{1}{2}}} \left( {i + l - \frac{1}{2},j} \right) - v_{r}^{{n + \frac{1}{2}}} \left( {i - l + \frac{1}{2},j} \right)} \right] \\ D_{z}^{ - } v_{r}^{n + 1/2} & = \mathop \sum \limits_{l = 1}^{L/2} a_{l}^{z} \left[ {v_{z}^{n + 1/2} \left( {i,j + l - 1/2} \right) - v_{z}^{n + 1/2} \left( {i,j - l + 1/2} \right)} \right] \\ D_{r}^{ + } v_{z}^{n + 1/2} & = \mathop \sum \limits_{l = 1}^{L/2} a_{l}^{r} \left[ {v_{z}^{n + 1/2} \left( {i + l + 1/2 \cdot j} \right) - \tau_{rz}^{n + 1/2} \left( {i - l + 3/2,j} \right)} \right] \\ D_{z}^{ + } v_{r}^{n + 1/2} & = \mathop \sum \limits_{l = 1}^{L/2} a_{l}^{z} \left[ {v_{r}^{n + 1/2} \left( {i.j + l + 1/2} \right) - \tau_{r}^{n + 1/2} \left( {i,j - l + 3/2} \right)} \right] \\ \end{aligned}$$

where Δt is the sampling interval in time; Δr and Δz are the spatial sampling intervals in the *r* and *z* directions, respectively; and ${v}_{r}$ and ${v}_{z}$ are the velocity components in the *r* and *z* directions, respectively. Such a difference format is advantageous because it uses a high-order difference operator between space and time. This way, high calculation accuracy is ensured, while a larger space step is used when possible to further improve the calculation efficiency^37^. Compared with the sound pressure–velocity differential format, this velocity-stress differential format does not require considering the interface between the various media. Thus, such an approach stands out with its remarkable advantages for simulating fracture-formation models.

**Figure 4:**
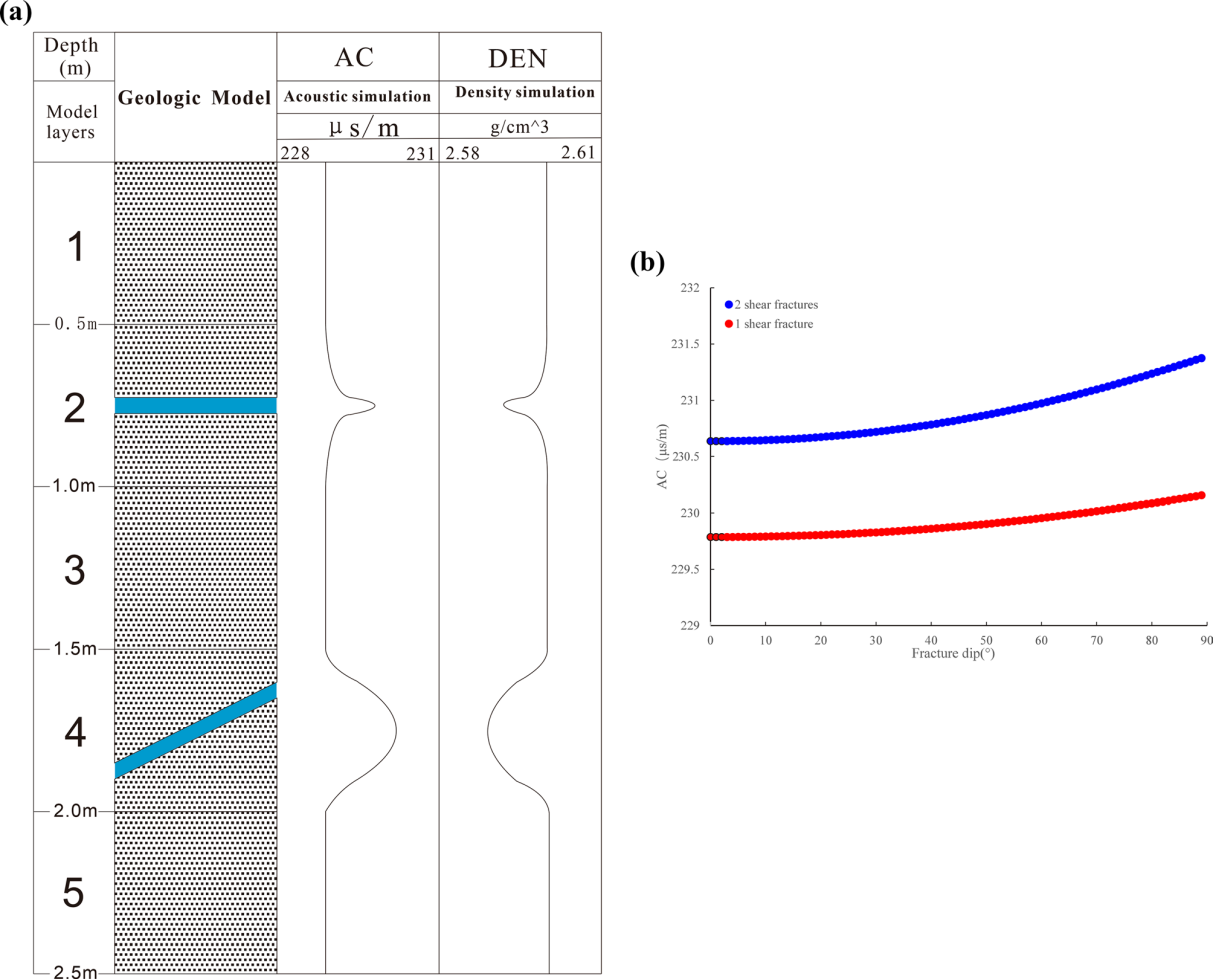
A geological model and response characteristics. (a) A geological model with five layers of tight quartz sandstone from top to bottom and physical models of two fractures, the fracture dip of the second layer is 0°, and the fracture dip of the fourth layer is 30°. (b) The response characteristics of the acoustic time difference of one and two fractures in the tight quartz sandstone with a fracture dip angle.

**Figure 5:**
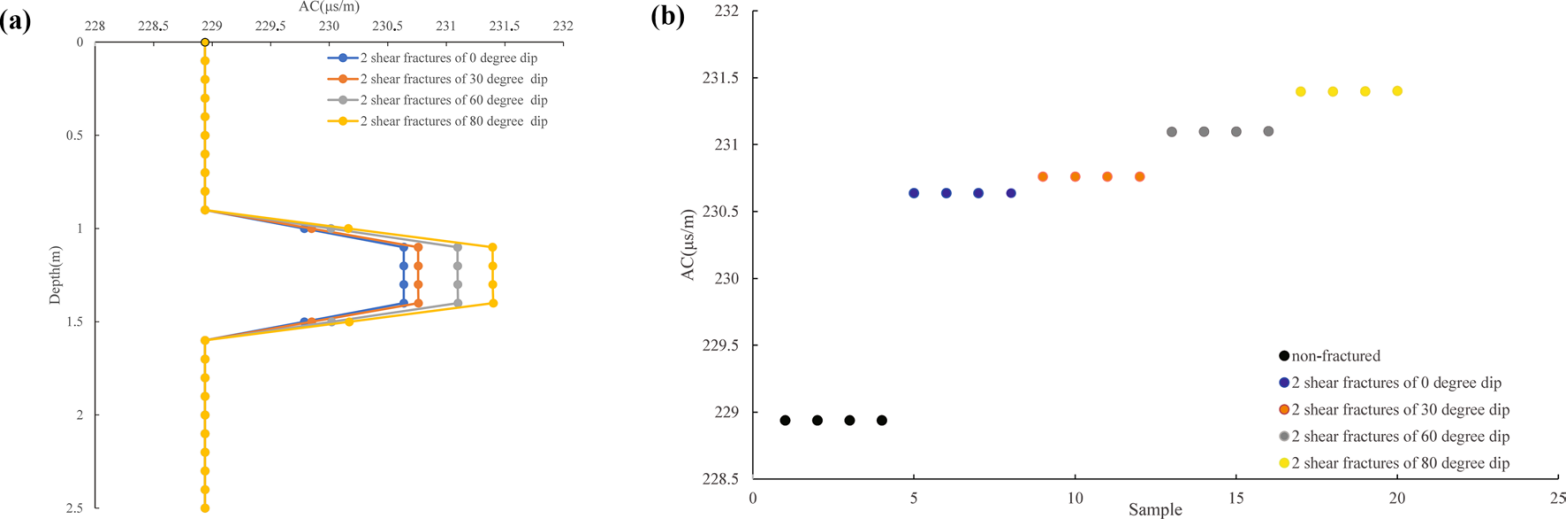
Response characteristics of the acoustic time difference between intact rock and different dip fractures. (**a**) Response characteristics of the acoustic time difference between the non-fracture development (0–0.9 m and 1.6–2.5 m) and fracture development sections (0.9–1.6 m), with four fracture dip angles: 0°, 30°, 60°, and 80° dips. (**b**) Response characteristics of the acoustic time difference between non-fractured sections and different dip fractures.

A geological model with five layers of tight quartz sandstone from top to bottom was constructed based on the geological characteristics of tight sandstone reservoirs in TX2 in the study area. Physical models of different fracture types were designed for the second and fourth layers. The fracture dip of the second and the fourth layer was 0° and 30°, respectively. No fractures were identified in other layers, as shown in Fig. 4a. The acoustic time difference for a unit length of tight quartz sandstone was 228.9377 μs/m, the density of tight quartz sandstone was 2.60 g/cm^3^, and fracture width was 1 mm. First, the propagation of acoustic waves in tight quartz sandstone with one and two fractures was simulated. As the fracture dip increased, the acoustic time difference proportionally increased. Consequently, the acoustic time difference response value of the two fractures clearly exceeded that of one fracture, as shown in Fig. 4b. As the fracture dip angle increased, the acoustic time difference response value also increased. Moreover, as the number of fractures increased, the acoustic time difference response value also increased. Note that the acoustic time difference response characteristics of non-fractured tight quartz sandstone were also simulated. In this context, no response change was identified, indicating no changes in the response of the acoustic time difference in the non-fractured tight quartz sandstone (Fig. 4a). The first, third, and fifth layers were non-fractured layers, and their acoustic time difference responses did not change. In Fig. 5a, 0.0–0.9 m and 1.6–2.5 m represent the non-fractured development intervals, and their acoustic time difference response did not change. However, 0.9 m to 1.6 m is the fracture development range, and the acoustic time difference response increased significantly. Moreover, as the fracture dip increased, the acoustic time difference also significantly increased. This pattern further suggests that the responses of the acoustic time difference to the occurrence of fractures and a lack of fractures in tight quartz sandstone were significantly different. The acoustic time difference can be used to identify fractured and intact sections. Figure 5b shows the distributions of the acoustic time difference for the non-fractured section and that with different fracture dip angles. The analysis of this data revealed a large difference between the acoustic time difference of the non-fractured section and the acoustic time difference of the fractured section. As the fracture dip increased, the acoustic time difference response also increased. Hence, the acoustic time difference responses to fractures with different dip angles were notably different, indicating that the acoustic time difference can be used to identify non-fractured and different types of fractures^38^.

**Figure 6:**
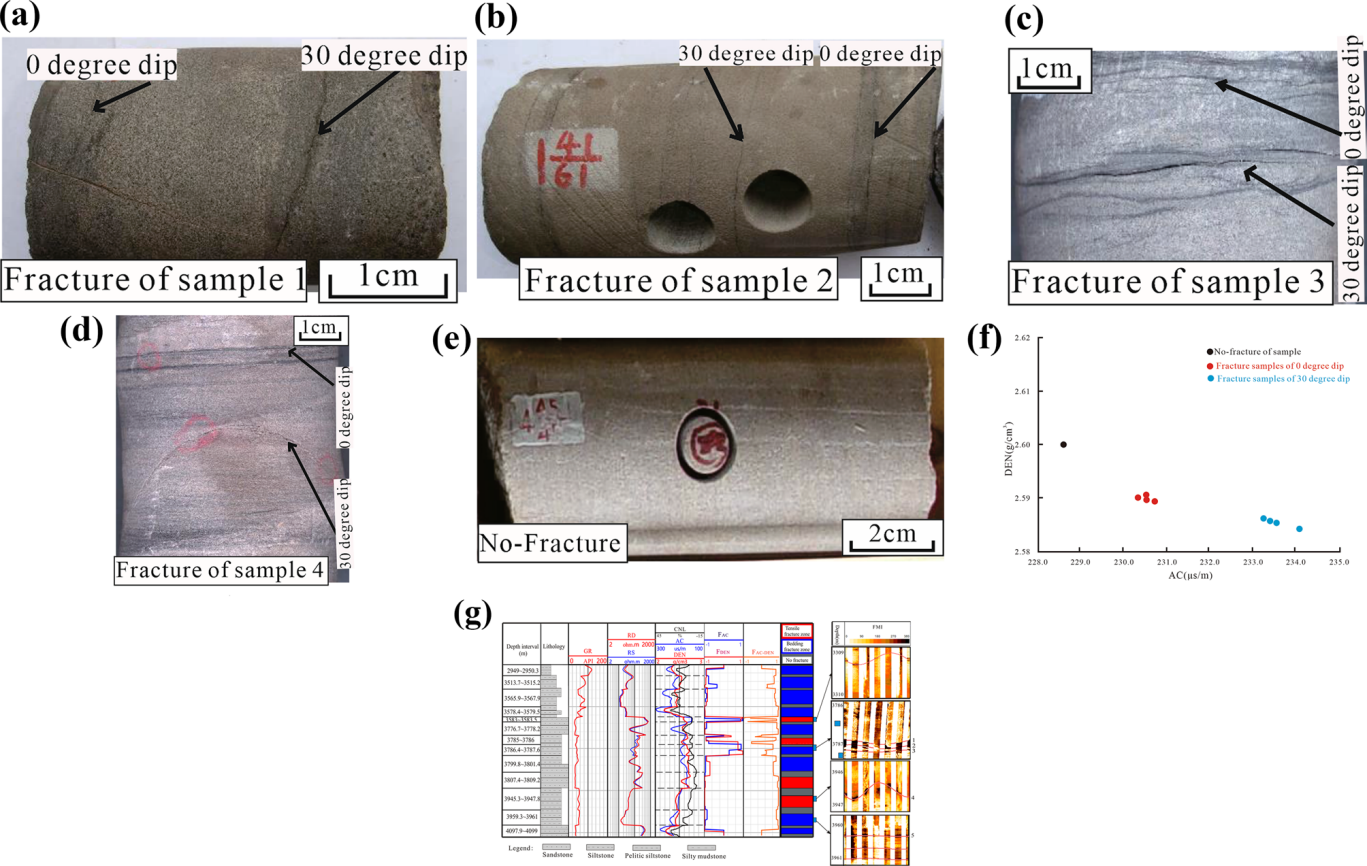
Core sample and actual log response characteristics of non-fractured rock and different dip fractures. (**a**) Fracture of sample 1. (**b**) Fracture of sample 2. (**c**) Fracture of sample 3. (**d**) Fracture of sample 4. (**e**) Non-fractured sample. (**f**) Acoustic-density response characteristic intersection diagram for fractures and non-fractures. (**f**) Acoustic-density response characteristic intersection diagram for fractures and non-fractures. (**g**) The red area is the tension fracture development zone, the blue area is the bedding fracture development zone, and the gray area is the intact zone. F_AC_ is the AC correlation coefficient, F_DEN_ is the DEN correlation coefficient, and F_AC-DEN_ is the AC-DEN correlation coefficient (compared with imaging results), showing that F_AC-DEN_ can adequately identify fracture zones. FMI is Formation MicroScanner Image.

Four typical core samples were preferentially acquired. In particular, the core samples were from the upper Triassic Xu 2nd member (T3X2) of the research area, as shown in Fig. 6a–e. Each sample has two types of fractures: a bedding fracture with a dip degree close to 0 and a low-angle fracture with a dip degree of about 30 degrees. The measured acoustic time difference and density of the samples are reported in Table 1. According to the results of core experiments (Table 1), the acoustic time difference and density of the tight quartz sandstone without fractures were 228.9377 μs/m and 2.60 g/cm^3^, respectively. When the fracture angle was 0°, i.e., horizontal, the measured acoustic time difference and density values for a fracture in sample 1, sample 2, sample 3, and sample 4 were 229.7870 μs/m and 2.593 g/cm^3^, 229.6094 μs/m and 2.592 g/cm^3^, 229.8739 μs/m and 2.596 g/cm^3^, and 229.7870 μs/m and 2.599 g/cm^3^, respectively. When the fracture angle was 30°, the measured acoustic time difference and density values for a fracture in sample 1, sample 2, sample 3, and sample 4 were 230.1754 μs/m and 2.5847 g/cm^3^, 234.6189 μs/m and 2.5837 g/cm^3^, 233.4312 μs/m and 2.5850 g/cm^3^, and 230.2864 μs/m and 2.5854 g/cm^3^, respectively. The measured response characteristic data are shown in Table 1. The acoustic-density response characteristic intersection diagram of fractures for four samples is shown in Fig. 6f. Analyzing Fig. 5 and 6f reveals that (1) the numerical simulation results are consistent with the core experimental measurements and (2) there is a clear response difference when fractures are present. When fractures are developed, the acoustic time difference increases significantly, and the density is expectedly reduced so that identifying fractures is relatively straightforward.

**Table 1:** The measured response characteristic data of different fractures and non-fractures in the same lithology sample.

Response characteristic value	Fracture dip	Fracture of sample 1	Fracture of sample 2	Fracture of sample 3	Fracture of sample 4	Numerical simulation of fractures	No-fracture
AC (μs/m)	0°	230.79	230.61	230.87	230.79	229.81	228.94
	30°	233.18	234.62	233.43	233.29	230.10	
DEN (g/cm^3^)	0°	2.59	2.59	2.60	2.59	2.59	2.6
	30°	2.58	2.58	2.58	2.58	2.58	

## Research on geomechanical log responses and fracture identification in tight sandstone

### Log curves are sensitive to the presence of fractures in tight sandstone

The analysis of the response characteristics of log curves to fractures is shown in Fig. 6g. The trend and form of the acoustic (AC) travel time and density (DEN) log curves exhibited regular changes, mainly manifested at the same location but with opposite trends, where one curve decreased, while the other increased. This pattern can also change depending on location in short temporal scales because they are affected by the different resolutions of the AC and DEN curves as well as the complexity of the local mineralogy and adjacent fractures. Although the neutron curve is fundamentally associated with the AC and DEN curves, the neutron curve trend in relation to the AC and DEN curves was unclear. In some cases, the CNL log response is similar to the DEN log response; however, the CNL log response is less sensitive to fractures than the DEN log response, especially in tight formations. Moreover, we found that the response of the dual laterolog to fractures in tight reservoirs was not significant. In addition, the AC and DEN log curves of fractures with different geological genesis exhibited the clearest and the most stable responses. The log response characteristics of the AC and DEN curves to fractures with different geological geneses indicate that the various types of fracture zones can be identified by estimating the characteristics of different DEN and AC log responses (Fig. 6g).

**Figure 7:**
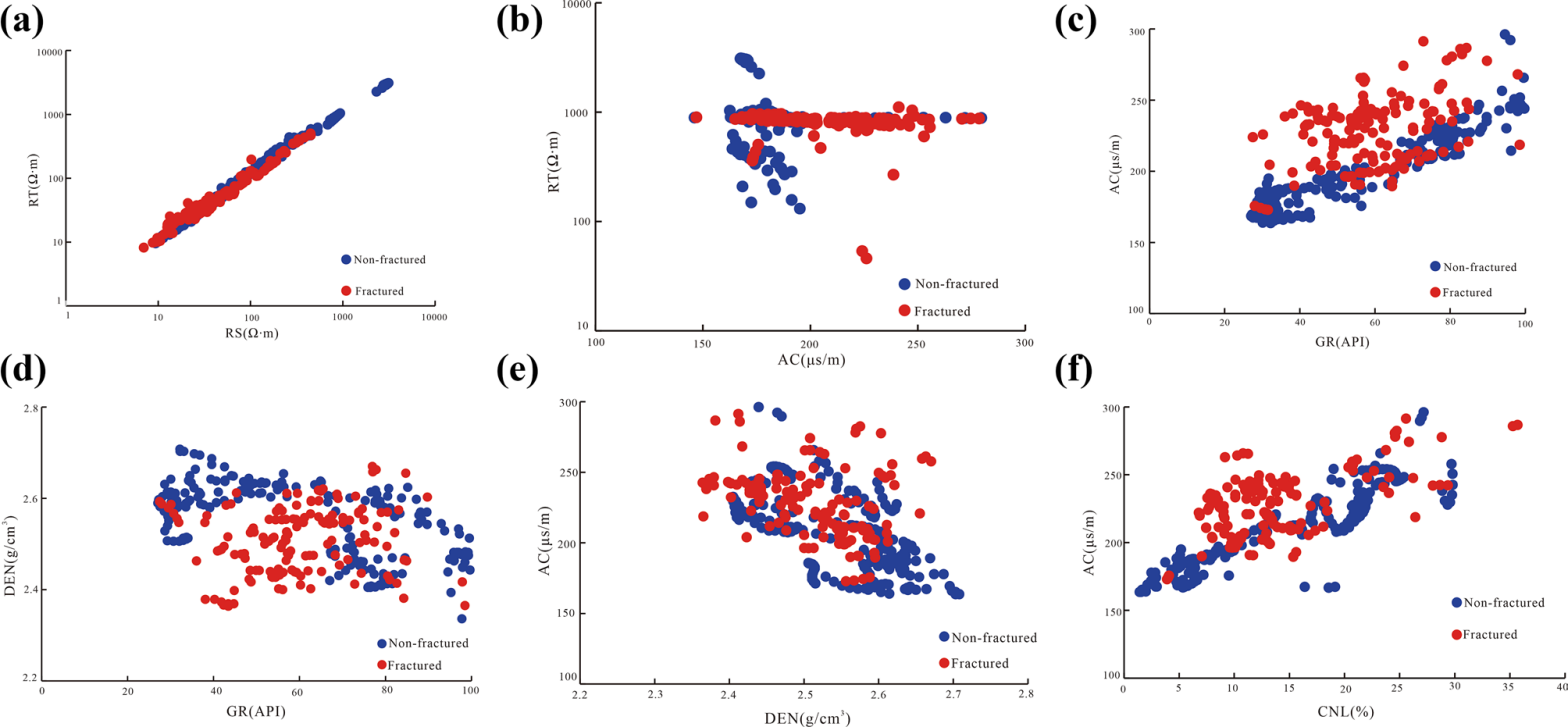
Logging sensitivity responses characteristics of fractured and intact rock.

We statistically compared the log response characteristics of sections with different fracture developments and found that the sensitivity of different log curves to fractured rocks varied. Figure 7 shows the log response diagram of a fracture. In particular, Fig. 7a shows a cross-sectional view of the deep lateral resistivity (RT) and shallow lateral resistivity (RS) data that reflect the areas with and without fracture development. Figure 7b illustrates a cross-sectional diagram of the acoustic time difference (AC) and the RT data, reflecting the areas with and without fracture development. Figure 7c,d display the natural gamma (GR) data that reflect the areas with and without fracture development and their corresponding acoustic time difference and compensation density (DEN) responses, respectively. Figure 7e,f show the cross-plots of the acoustic time difference data, reflecting the areas with and without fracture development and their corresponding compensation density and compensated neutron (CNL) responses, respectively. The red data points in Fig. 7 are the log response values that mark the presence of a fracture, and the blue data points are the log response values, marking the absence of a fracture. The comparison demonstrated that the dual laterolog resistivity cross-plot of data collected in fractured and intact rock exhibited a linear relationship (Fig. 7a). Moreover, the responses from the areas with and without fractures were somewhat indistinguishable, and the data points overlapped. However, some differences in the resistivity and acoustic time responses of the areas with and without fractures were discerned, as shown in Fig. 7b, but most of the data points overlapped. It is, therefore, reasonable to suggest that RT is not sensitive to the presence of fractures and cannot be used to characterize fractures. The cross-plots (Fig. 7c–f) revealed a clear distinction between the data points representing the fractured and intact rock. In particular, the data points of the corresponding log responses significantly differed. These log responses were independent of each other. In particular, the difference in the log response data distribution between the fractured rock and intact rock was more conspicuous in Fig. 7c (acoustic time difference and natural gamma cross-plot) and Fig. 7d (density and natural gamma cross-plot). In these figures, the log response data points (red) of the fracture were closely clustered, and the distribution of the log response data points (blue) in the intact rock did not overlap. This finding indicates that the acoustic time difference, density, and natural gamma are very sensitive to the presence of fractures and can be used to characterize different fractures.

**Figure 8:**
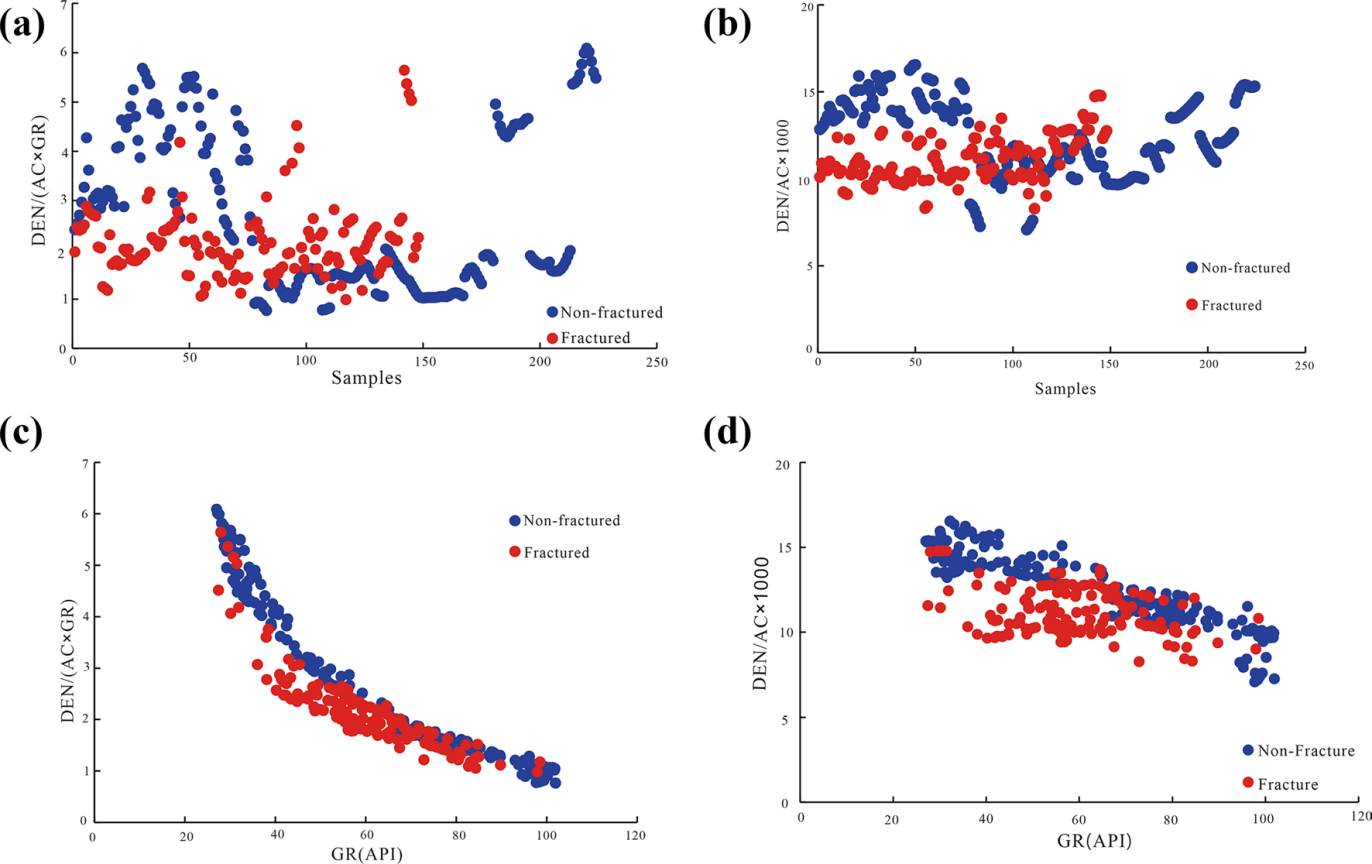
Logging sensitivity responses characteristics of fractured and intact rock after processing well-logging data.

Moreover, Fig. 7c–e, clearly demonstrate that despite the natural gamma, acoustic time difference and density are sensitive to the fractures, and there is a clear distinction among the responses. The acoustic wave time difference and density data from fractured and intact rock overlap in a cross-plot. Therefore, the acoustic time difference and density are relatively insensitive to the presence of a fracture, thereby indicating that the efficiency of fracture identification and characterization is uncertain. Ideally, this uncertainty should be eliminated while selectively retaining the signal characteristics of fracture information. Our results hinted that we could eliminate the mud signal in logging data, retaining the rock skeleton and only fracture information. To this end, the petrophysical volume response equation was established, while the volume and response value of each section were iteratively solved. Furthermore, the logging signal contribution value of the mud portion was filtered out from the logging signal. As a result, only the response value of the skeleton and the fracture–pore component was retained^2,39^_._ Fracture-sensitive logging data were processed to amplify the difference between the responses of fractured and intact rock (Fig. 8) according to the log response characteristics of the data points from the fractured rock (Fig. 7c–e). Although the sensitivity of the log curves to the presence of a fracture slightly varied, we confirmed that the acoustic time difference, density, and natural gamma log curves were sensitive to fractures (Fig. 8).

### Log and rock physical response characteristics of tight sandstone fractures

**Figure 9:**
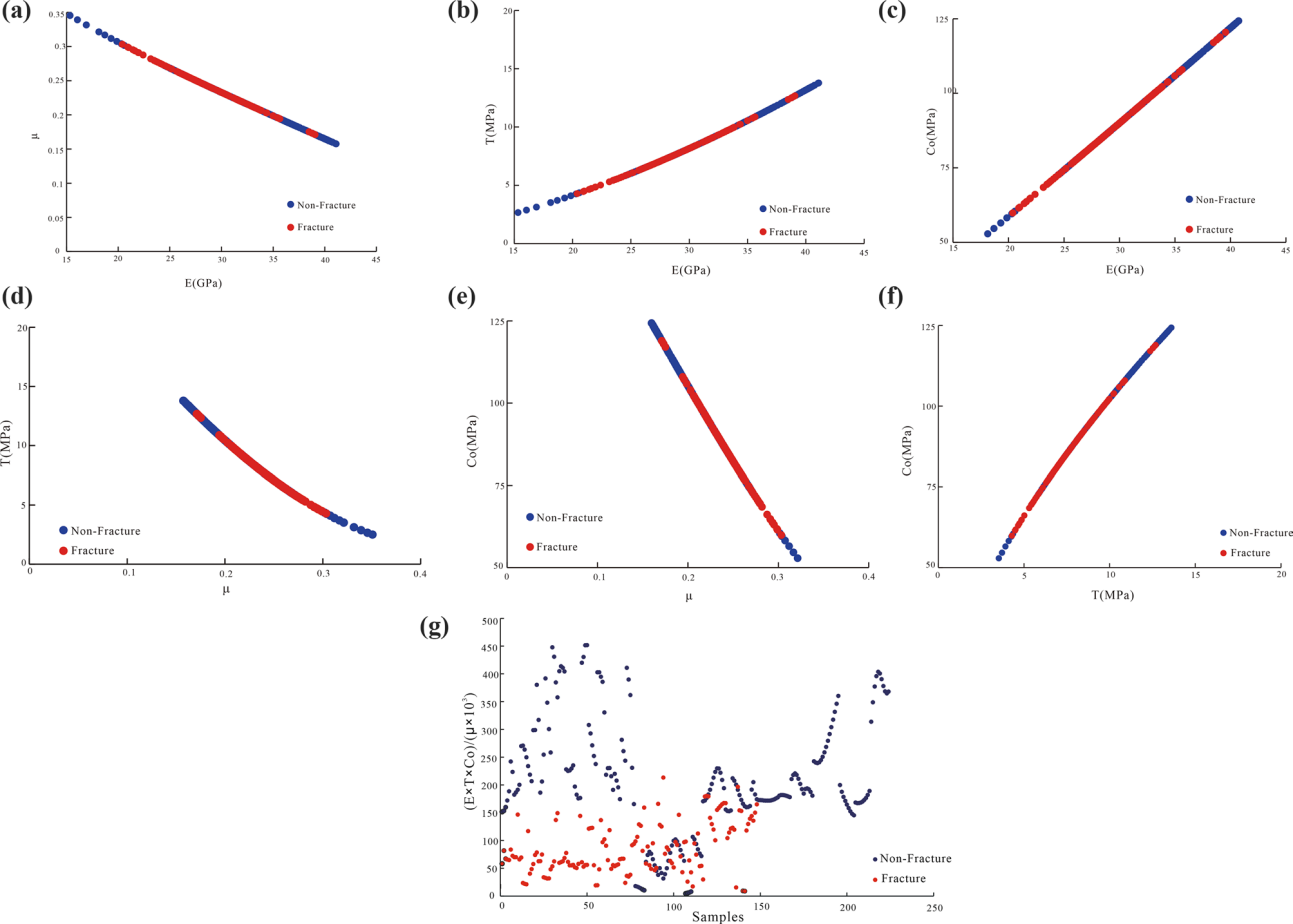
Physical response characteristics of fractured and non-fractured rock, including Young’s modulus (E), tensile strength (T), compressive strength (C_o_), and Poisson’s ratio (μ).

The logging data and rock physical parameters of the tight sandstone fractures were analyzed using curves sensitive to different fractures. After the differential processing of the log response data of the fractured and intact rocks, the log response characteristics of the fracture (Fig. 8) as well as its geomechanical parameters (Fig. 9) were determined. The analysis of the log responses to fractures (Fig. 7) demonstrated that AC, DEN, and GR were the most sensitive to the presence of fractures. The fractures revealed various log responses, but some overlap was discerned (Fig. 7). To outline the differences among the overlapping data trends, we sought apparent differences between the data of the fractured and intact rock. In this process, we first normalized the data from the sensitive logs AC, DEN, and GR. To retain the data authenticity without changing the amplitude and trend of the data, log data were divided by the maximum value of the data, obtaining normalized values. Next, using the ratio method, we utilized the change in the relationship between fractured and intact rock to obtain the ratio parameters DEN/(AC × GR) and DEN/AC × 1000. Specifically, DEN/(AC × GR) and DEN/AC × 1000 were derived from the change trend of sensitive logging data between the fractured and intact rock. Notably, these two ratios are highly sensitive to the presence of fractures and can be used for efficient delineation of fractured rocks from intact rocks. On this basis, cross-plots of the fractured rock and intact rock response characteristics were established (Fig. 8).

The results of the log response characteristics analysis of fractured rock data are shown in Fig. 8. First, the ordinates, shown in Fig. 8a,b, are the ratio parameters DEN/(AC × GR) and DEN/AC × 1,000, respectively, while the abscissa is the number of sample points. The difference in response between the fractured and intact rock shown in Fig. 8b was less remarkable than that in Fig. 8a. Particularly, the ratio parameters of the intact rock (blue data points) in Fig. 8b were significantly higher than those of the fractured rock (red data points), with only a few overlapping data points. Therefore, the ratio DEN/AC × 1,000 can be deemed more suitable than DEN/(AC × GR) for characterizing the response characteristics and differences between fractured and intact rocks. Second, the ordinates in Fig. 8c,d are the ratio parameters DEN/(AC × GR) and DEN/AC × 1,000, respectively, while the abscissa is the GR. The difference in the response between the fractured rock and intact rock shown in Fig. 8d was less remarkable than that in Fig. 8c. In other words, the ratio parameter DEN/AC × 1000 of the intact rock data (blue) in Fig. 8d was significantly higher than that of the fractured rock data. The difference between the two, as well as the boundaries, were both clear. The ratio parameter DEN/(AC × GR) of the intact rock data shown in Fig. 8c was also significantly higher than that of the fractured rock data. However, the GR values, attributed to fractured and intact rock, overlapped between 60 and 85 API. Thus, the boundaries were rather vague. Third, the ratio of the DEN/AC × 1000 fracture to that of intact rock decreased with increasing GR. In particular, the ratio parameter DEN/AC × 1000 inversely correlated with GR. Notably, the fracture and intact rock were clearly distinguished, with a clear boundary, confirming that AC, DEN, and GR are sensitive to fractures. Moreover, these results indicate that fractures can be identified and characterized by the differences in the responses of the ratio parameter DEN/AC × 1000 and GR.

Triaxial rock mechanics experiments were performed on core samples, and extensive data about geomechanical parameters were obtained (Table 2 and Fig. 9). Table 2 summarizes the measured geomechanical parameters of fractured and non-fractured tight sandstone, including some rock physical parameters sensitive to the presence of fractures: Young’s modulus (E), tensile strength (T), compressive strength (Co), and Poisson’s ratio (μ). The analysis of the physical parameters of fracture-sensitive rock is shown in Fig. 9. As seen, Young’s modulus (E), tensile strength (T), compressive strength (C_o_), and Poisson’s ratio (μ) were sensitive to the presence of fractures. Moreover, the fractured and intact rocks exhibited clear differences in their geomechanical parameter responses. The fractured rock data were distributed in a specific rock physical response interval but overlapped with the intact rock data. Second, the E, T, μ, and C_o_ values, related to the fracture featured clear response intervals and differences. The Young’s modulus (E) response to the fractured tight sandstone was distributed between 20 and 35.5 GPa, the tensile strength (T) response was clustered between 4 and 10.3 MPa, and the compressive strength (C_o_) response was distributed between 59.8 and 109.8 MPa. The Poisson’s ratio (μ) response ranged from 0.2 to 0.3. Third, Young’s modulus (E) response of intact, tight sandstone was distributed between 15 and 20 GPa and 30 and 41 GPa, and the tensile strength (T) response was identified between 2.6 and 4.2 MPa and 8.3 and 13.8 MPa. The compressive strength (C_o_) response was clustered between 52 and 60 MPa and 86 and 125 MPa, and the Poisson’s ratio (μ) response was distributed between 0.15 and 0.2 and 0.3 and 0.35. Fourth, the E and T curves, E and Co curves, as well as T and C_o_ curves related to the fractured and intact rocks were significantly positively correlated. However, we discerned a significant negative correlation between the E and μ curves, μ and T curves, and μ and C_o_ curves. The above comparison suggests that the responses of the E, T, and C_o_ parameters corresponding to the fractured rock and intact rock were somewhat the same, while the μ response was the same between rock types but opposite to those of the other parameters. The four parameters found to be the most sensitive to the presence of a fracture can all be utilized to efficiently characterize the fracture parameters.

**Table 2:** Rock physical response values of fractured and nonfractured tight sandstone.

Rock physical response values		Fracture in tight sandstone	No-fracture in sandstone
E (GPa)	Min	20.0	< 20.0 and > 35.5
	Max	35.5	
	Average	27.7	
T (MPa)	Min	4.0	< 4.0 and > 10.3
	Max	10.3	
	Average	7.1	
C_o_ (MPa)	Min	59.8	< 59.8 and > 109.8
	Max	109.8	
	Average	84.8	
μ	Min	0.2	< 0.2 and > 0.3
	Max	0.3	
	Average	0.25	

## Identification method of tight sandstone fractures

AC and DEN logs are sensitive to the presence of fractures. The fractured and intact rock plots represented independent distribution intervals along the AC and DEN curves, and the corresponding AC and DEN responses demonstrated a significant negative correlation. The DEN/AC × 1000 values of the fracture and intact rock decreased with increasing GR, namely, the ratio parameter DEN/AC × 1000 negatively correlated with GR. The fractured rock data were clearly different from the intact rock data. The boundaries between the two were somewhat clear (Fig. 8d). This property was used to construct an eigenvalue function EvF that can distinguish between fractured and intact rocks, as shown in Eq. (5), i.e., Eq. (5) is an eigenvalue function EvF constructed for dividing fractures and non-fractures^38^.

5 $${\text{EvF}} = \frac{{\left( {\frac{{{\text{DEN}}}}{{{\text{DEN}}_{{{\text{MAX}}}} }}} \right)}}{{\left( {\frac{{{\text{AC}}}}{{{\text{AC}}_{{{\text{MAX}}}} }}} \right)}} = \frac{{{\text{DEN}}}}{{{\text{AC}}}} \times 10^{3}$$

**Figure 10:**
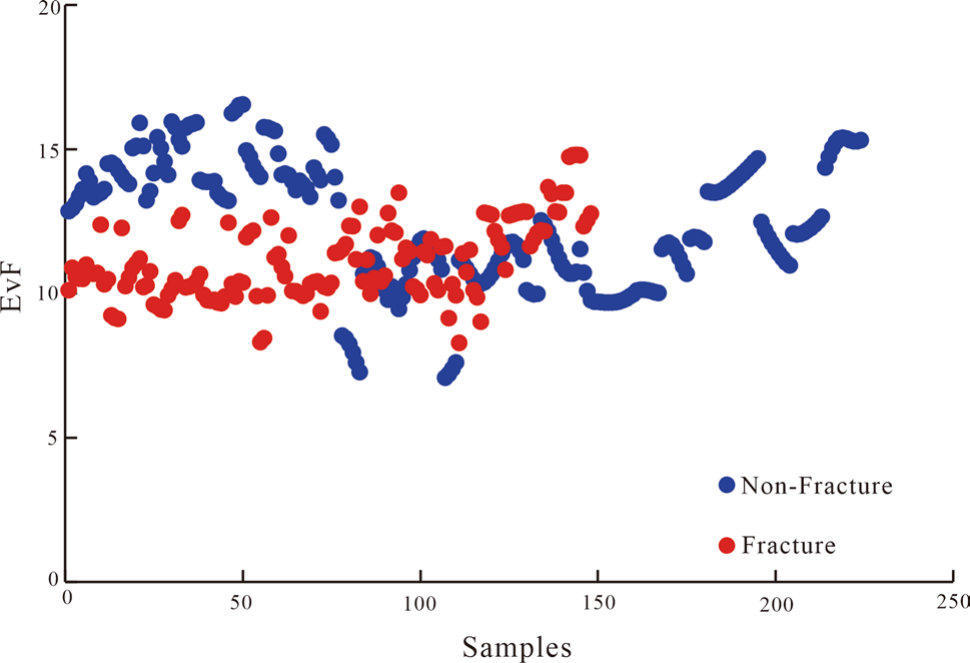
The range of the EvF distribution of fractured and intact rock.

The EvF values of the fractured rock and intact rock data were calculated with Eq. (5) to obtain the range of the EvF distribution (Fig. 10). We further analyzed their distribution ranges and found that the EvF range of fractured rock had a small span, mainly ranging between 9 and 13. However, the span of EvF range distribution of intact rock was large and mainly distributed between 7 and 16. Second, we identified a partial overlap between the EvF ranges of fractured and intact rock. However, the fractured rocks could not be distinguished from intact rocks only by considering the EvF range. Thus, more attribute parameters were required to distinguish fractured rocks from intact rocks.

**Figure 11:**
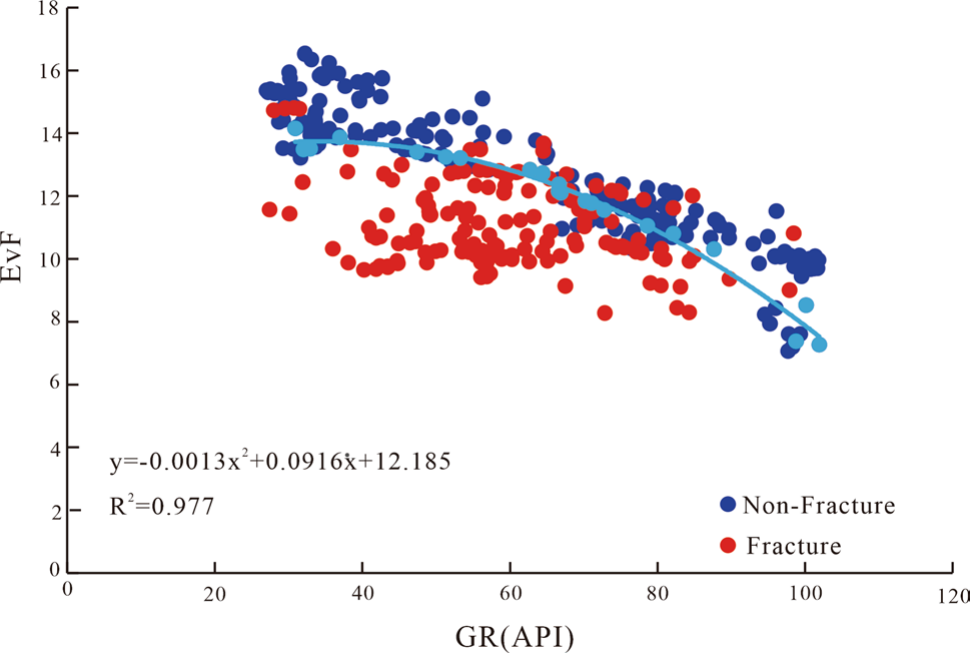
Introducing the GR value to the eigenvalue function EvF further enhances distinguishing fractured and intact rock responses.

To this end, we analyzed the AC and GR cross-plots of the fractured rock data (Figs. 7c,d and 8d). The analysis of the fractured rock data points demonstrated that the AC and GR values of the fractured and intact rocks differed significantly and positively correlated; however, the slopes of the AC-GR curves were significantly different. Introducing the GR value to the eigenvalue function, EvF, allowed distinguishing the fractured rock and intact rock responses, as shown in Fig. 11. Although Fig. 11 illustrates a very wide interval of the GR distribution (generally 27–100 API), the GR distribution interval of fractured rock was generally < 90 API. Second, we found that the intact rock data plot was relatively high, but the fractured rock data plot was relatively low in the cross-plot of EvF and GR. A clear dividing line between the two distributions was identified and can be described by Eq. (6), i.e., based on the distribution of fractured and non-fractured rock, Eq. (6) is the boundary function constructed to distinguish fractured and intact rock^38^. Notably, this division can be used to distinguish between fractured and intact rocks.

6 $${\text{EvBL}} = - 0.0013 \times {\text{GR}}^{2} + 0.0916 \times {\text{GR}} + 12.185$$

The eigenvalue (EvBL) standard (i.e., the criterion) was established according to the eigenvalue function EvF. The EvF value was generally distributed in the interval of 9–13, reflecting a fracture. In particular, when the calculated EvF value was less than the limit value EvBL (EvF < EvBL), it was assumed to indicate fractured rock. When the calculated EvF value was greater than the threshold value EvBL (EvF > EvBL), it was assumed to indicate intact rock. The fractures were better distinguished through the EvF eigenvalue function identification model.

We identified fractures using logging data as stress is the leading cause of fractures, and geomechanical parameters can be used as effective parameters for identifying fractures. Figure 9 shows the fracture-sensitive geomechanical parameters. The physical parameters E, T, C_o_, and μ of the rocks were sensitive to fractures. We identified clear differences in the geomechanical parameters between fractured and intact rock because the corresponding E, T, μ, and C_o_ of fractures exhibited clear response intervals and differences. Therefore, it can be suggested that using these characteristics can amplify the differences between fractured and intact rock responses. We further attempted to evaluate this result iteratively. To this end, we used rock physical parameters to construct the eigenvalue function MvF, which distinguishes fractured rock from intact rock, as shown below:

7 $${\text{MvF}} = \frac{{\left( {\frac{{\text{E}}}{{{\text{E}}_{{{\text{MAX}}}} }}} \right) \times \left( {\frac{{\text{T}}}{{{\text{T}}_{{{\text{MAX}}}} }}} \right) \times \left( {\frac{{{\text{Co}}}}{{{\text{Co}}_{{{\text{MAX}}}} }}} \right)}}{{\left( {\frac{\mu }{{\mu_{{{\text{MAX}}}} }}} \right)}} = \frac{{{\text{E}} \times {\text{T}} \times {\text{Co}}}}{\mu } \times 10^{ - 3}$$

In Eq. (7), E is Young’s modulus, T is the tensile strength, Co is the compressive strength, and μ is the Poisson’s ratio.

**Figure 12:**
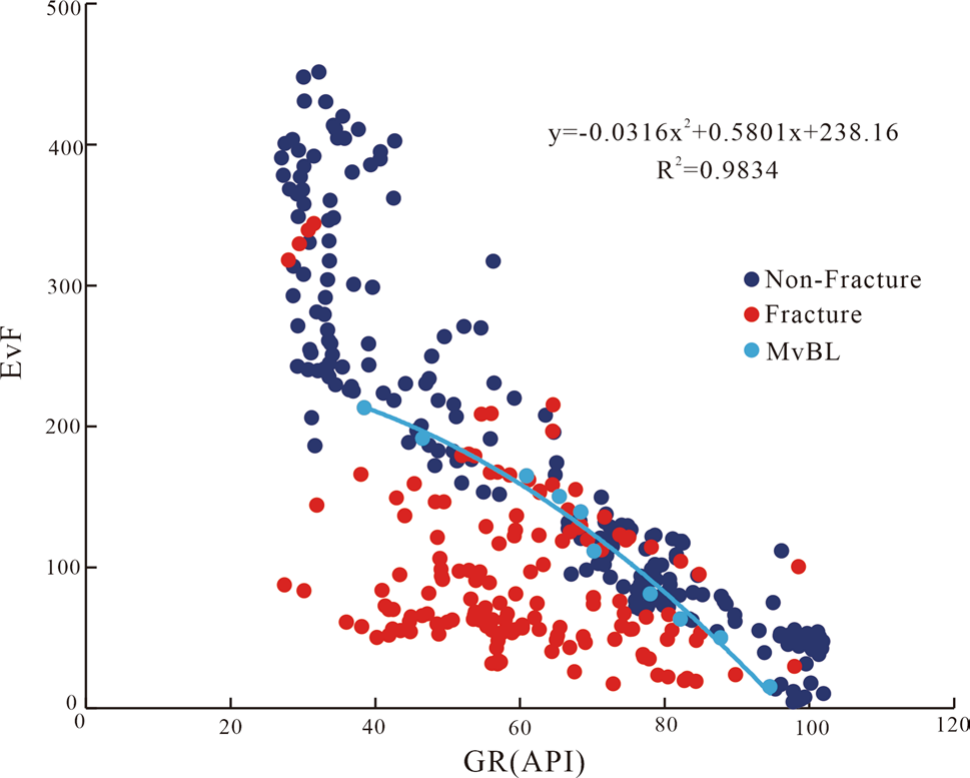
MvF and GR values have significantly different distributions between the fractured and intact rock.

Equation (7) was used to calculate the MvF values of rocks with fractures of different genesis to obtain their MvF distributions (Fig. 12). The analysis of their distribution ranges demonstrated that both the MvF and GR values significantly negatively correlated between the fractured and intact rock. The GR value distribution range of intact rock was large, mainly ranging between 30 and 100 API, while that of fractured rock was somewhat narrow (< 90 API). The GR distribution interval was used as a reference condition to discern between fractures. Second, the MvF and GR cross-plots, as well as the intact distribution interval plots, were relatively high, and the fractured distribution plots were relatively low. We identified a clear border between the two distributions that can be used to distinguish between fractured and intact rocks. This dividing line is described by Eq. (8).

8 $${\text{MvBL}} = - 0.0316 \times {\text{GR}}^{2} + 0.5801 \times {\text{GR}} + 238.16$$

The characterization value (MvBL) standard, namely, the criterion for identifying fractures, was established according to the eigenvalue function MvF. The MvF value of the fracture was generally distributed in the interval of 30 − 150. Note that when the calculated MvF value was below the limit value MvBL (MvF < MvBL), it was assumed that a fractured rock was identified. When the calculated MvF value exceeded the threshold value MvBL (MvF > MvBL), it was assumed that an intact rock was identified. Overall, the fractures were better distinguished when using the MvF eigenvalue function for their identification.

## Case study

**Figure 13:**
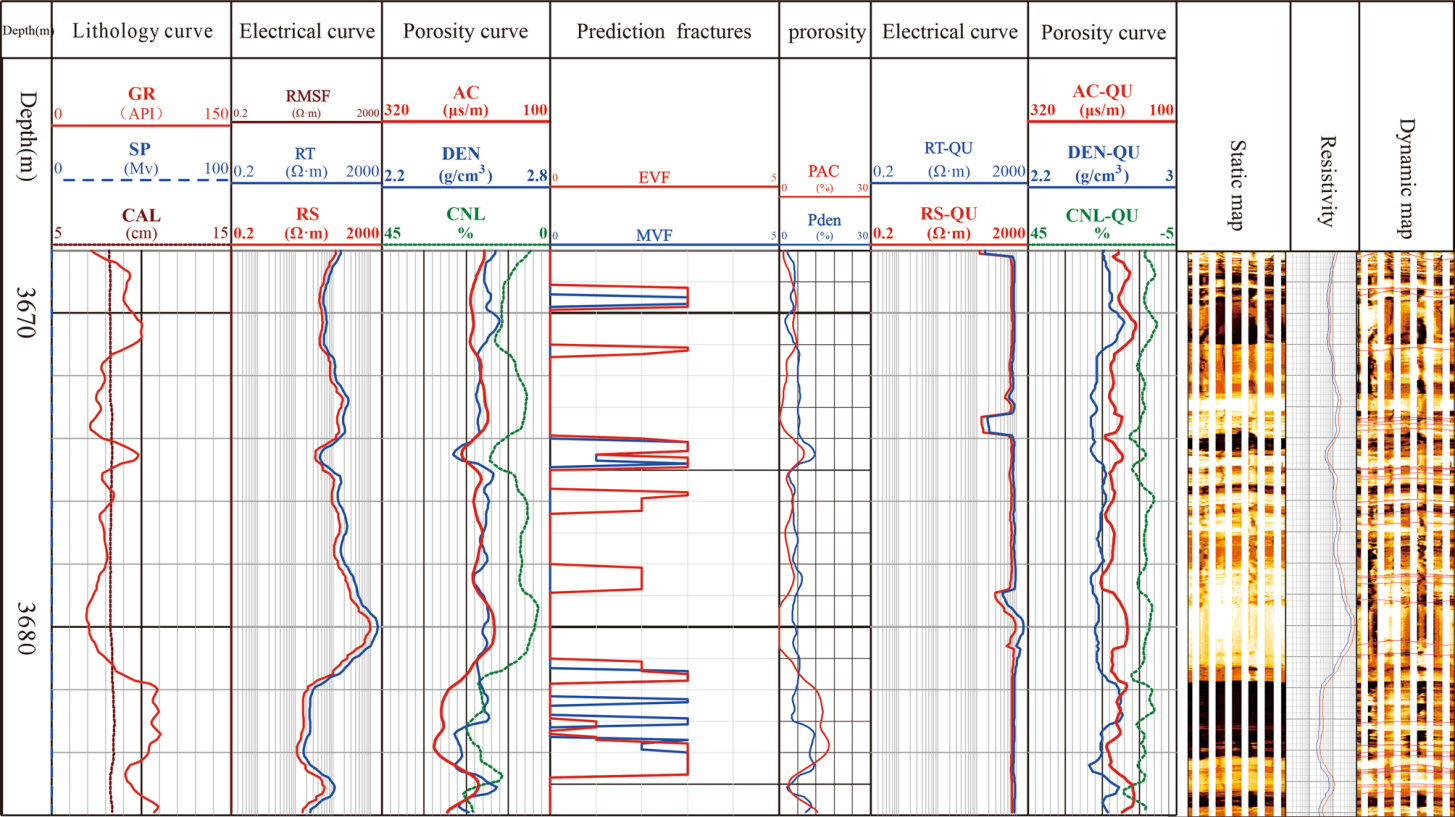
Comparing prediction results of fractures in Well XC25 (3668–3684 m) and the actual imaging log.

By applying the eigenvalue function model EvF and MvF, shown in Eqs. (5) and (7), respectively, we estimated a section of tight sandstone well XC25 to be 3668–3684 m deep. The two eigenvalue function values were extracted, indicating that the fracture development of this section was within the 3668–3684 m range of the XC25 well. This estimate was obtained according to the fracture criterion of the characteristic value (Fig. 13). We further compared discriminant results with the fractures developed in the electrical image. The comparison demonstrated that the fracture discrimination results between the eigenvalue function EvF and eigenvalue function MvF were consistent, effectively revealing the response characteristics of fractured rock. Second, a section with fracture development was identified in the image, and the eigenvalue function indicated that the location of this section resonated with that in the image log. Moreover, the eigenvalue function results were consistent with the actual results, indicating the efficiency of the constructed eigenvalue function discriminant model in this case study (Fig. 13).

## Discussion

**Figure 14:**
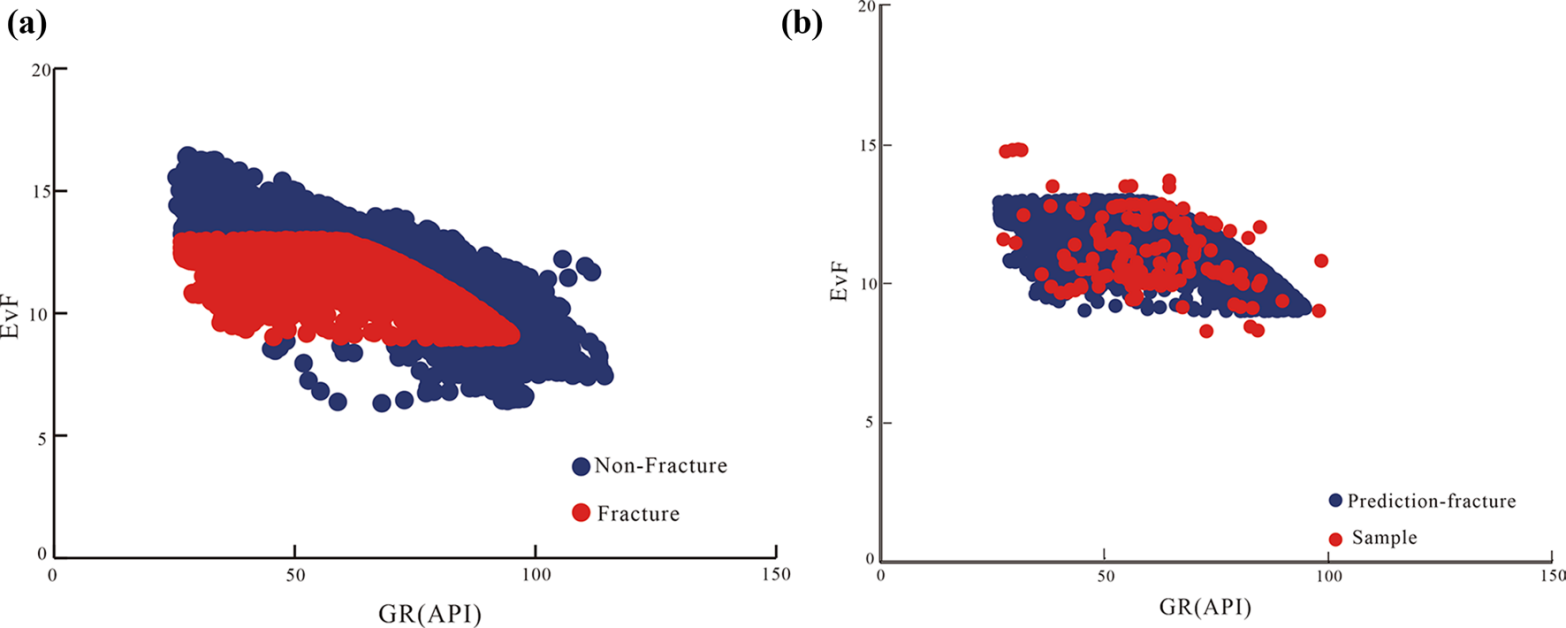
The predicting fracture development data points by EvF are consistent with the data points of fracture samples.

The eigenvalue function identification model EvF was further applied to identify fractures and sections of fracture development in the XC25 well (XU four sections). Moreover, we predicted the distribution of the data points related to these fractures (Fig. 14a). Specifically, the prediction indicated that the fracture development data points were perfectly consistent with the fracture sample data points (Fig. 14b). Furthermore, by analyzing Fig. 14a,b, we found that the predicted characteristic value of EvF corresponding to the fracture development data points showed a distinct response interval (the EvF value was 9–13) that significantly differed from that of EvF values of intact rock data points. Second, the prediction showed that the data points of fracture development sections and intact rock exhibited distinct responses, which facilitated their quantitative distinction. The predicted fracture development data points were in line with the distribution of the fracture sample data points. The EvF model was efficient when applied to the XC25 well, indicating the high accuracy of the eigenvalue function.

**Figure 15:**
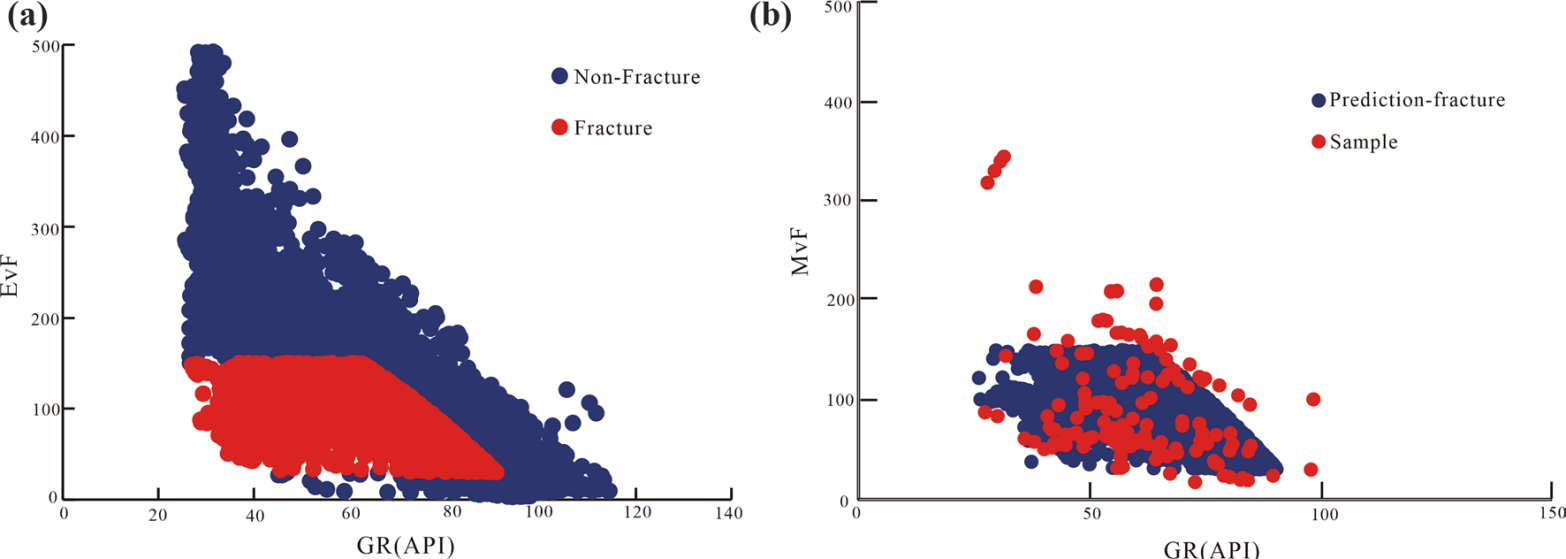
The predicting fracture development data points by MvF are consistent with the data points of fracture samples.

The identification model MvF was also used to identify fractures and sections of fracture development in the same section of the XC25 well (XU four-section) and to predict the distribution of the fracture development data points (Fig. 15a). The prediction indicated that the fracture development data points were consistent with the fracture sample data points (Fig. 15b). Furthermore, Fig. 15a,b show that the predicted characteristic value of EvF corresponding to the fracture development points exhibited a clear response interval (the MvF value was 30–150), which significantly differed from that of MvF values of intact rock data points. Moreover, it was predicted that the data points of fracture development sections and intact rock exhibited apparently unique responses, rendering their distinction relatively simple. Lastly, the predicted fracture development data points were consistent with the distribution of the fracture sample data points. Overall, the model was efficient for the XC25 well, thereby suggesting the high accuracy of the eigenvalue function of the MvF model.

## Conclusions

We analyzed numerical simulation results and log response characteristics of tight sandstone fractures, focusing on the log response characteristics and differences after removing the mud signal. The acoustic wave and density were very sensitive to the presence of fractures. The conventional logging response characteristics and response differences between fractures and non-fractures were clarified. In particular, with an increase in the fracture dip angle, the acoustic time difference increased, and the density decreased.

Rock physical response characteristics of fractured and intact rock demonstrated that E, T, C_o_, and μ were sensitive to the presence of fractures. Fractured and intact rocks exhibited clear differences in their geomechanical parameters, i.e., E, T, and C_o_ of fractures decreased with increasing μ, and the corresponding responses were distributed in different geomechanical response intervals.

Lastly, the logging eigenvalue method (EvF model) and rock physical eigenvalue method (MvF model) were established. The two methods were applied to actual wells to identify fractures. Most notably, the identification yield of the EvF model outperformed that of the MvF model.

## Data Availability

The datasets used and/or analyzed during the current study are available from the corresponding author on reasonable request.
